# Human Glial Cells as Innovative Targets for the Therapy of Central Nervous System Pathologies

**DOI:** 10.3390/cells13070606

**Published:** 2024-03-30

**Authors:** Giulia Magni, Benedetta Riboldi, Stefania Ceruti

**Affiliations:** Laboratory of Pain Therapy and Neuroimmunology, Department of Pharmacological and Biomolecular Sciences, Università degli Studi di Milano, via Balzaretti, 9, 20133 Milan, Italy; giulia.magni@unimi.it (G.M.); benedetta.riboldi@unimi.it (B.R.)

**Keywords:** astrocytes, microglia, iPSCs, organoids, neurodegeneration, neuroinflammation

## Abstract

*In vitro* and preclinical *in vivo* research in the last 35 years has clearly highlighted the crucial physiopathological role of glial cells, namely astrocytes/microglia/oligodendrocytes and satellite glial cells/Schwann cells in the central and peripheral nervous system, respectively. Several possible pharmacological targets to various neurodegenerative disorders and painful conditions have therefore been successfully identified, including receptors and enzymes, and mediators of neuroinflammation. However, the translation of these promising data to a clinical setting is often hampered by both technical and biological difficulties, making it necessary to perform experiments on human cells and models of the various diseases. In this review we will, therefore, summarize the most relevant data on the contribution of glial cells to human pathologies and on their possible pharmacological modulation based on data obtained in post-mortem tissues and in iPSC-derived human brain cells and organoids. The possibility of an *in vivo* visualization of glia reaction to neuroinflammation in patients will be also discussed.

## 1. Heterogeneity and Roles of Glial Cells in Neurodegenerative Pathologies of the Central Nervous System

When glial cells were first discovered, they were considered as “passive” cell populations with merely structural and supportive functions to sustain neuronal cells; however, as research in the field progressed, their heterogeneity and multifunctional properties progressively emerged. It is worth mentioning that, although the glia family includes a larger number of cell populations than those discussed in this review (i.e., oligodendrocytes, pericytes, Schwann cells, etc.), in this review we will only focus on those giving a recognized active contribution to central nervous system (CNS) damage, neurodegeneration and neuroinflammation, with some hints on their role in painful conditions.

In the CNS, microglia represent a highly dynamic and plastic cell population. Due to the presence of a complex “sensome”, i.e., a series of surface receptors that allow them to patrol the surrounding environment, microglia are capable of reacting to any possible modification by changing their functional state. Different cellular states are characterized by changes in morphology, ultrastructure and molecular profile as well as in motility, function and the expression of specific markers. Two distinct polarization profiles were initially described for microglia: a pro-inflammatory one (M1), responsible for the production of cytokines, chemokines and metabolites involved in neuroinflammation and neurodegeneration, and an immunoregulatory one (M2), implicated in neuroprotection and damage repair processes. However, today this classification is outdated, and it is preferred to refer to highly dynamic microglia that exhibit multivariate functional, morphological and metabolic states [1].

Astrocytes are essential for the maintenance of the neuronal environment and take part in many homeostatic processes. As for microglia, their reactivity is highly heterogeneous, with different types of damage inducing different subtypes of reactive astrocytes. In fact, they can be activated to the pro-inflammatory neurotoxic A1 phenotype through several mechanisms that are, in many cases, triggered and mediated by activated microglia, thus contributing to neurodegeneration, or to the A2 phenotype as a protective mechanism allowing recovery from damage [2]. Additionally, heterogeneous populations of astrocytes have been discovered depending on the brain area, thus adding further complexity to their physiological and pathological roles [3]. Astrocytes are also key components of the so-called “glymphatic” system which allows for the correct clearance of waste and toxic substances from the brain parenchyma and whose compromission is emerging as a key element contributing to several brain pathologies, as recently reviewed in [4].

In the peripheral nervous system (PNS), satellite glial cells (SGCs) residing in sensory ganglia are often compared to astrocytes due to their many shared features, including the trophic support of neurons, control of extracellular glutamate levels, production of cytokines and chemokines and their switch to an activated state following damage of various origins [5]. In fact, SGCs also display morphological and functional heterogeneity, although their role has been much more clearly delineated in pain transmission than in neurodegeneration [6].

Conversely, it is well recognized that CNS glia actively participate in the development and progression of several neurodegenerative disorders (e.g., Alzheimer’s disease (AD), Parkinson disease (PD), multiple sclerosis (MS), and amyotrophic lateral sclerosis (ALS)), and in the maintenance of chronic pain. Neurodegeneration refers to the chronic and progressive loss of neurons in the brain and spinal cord [7], and one of its main histological features consists of protein aggregation in specific brain regions [8]. For example, AD is characterized by the formation of amyloid-beta (Aβ) plaques and neurofibrillary tangles that cause memory loss and cognitive decline [9], while PD is linked to the selective death of dopaminergic neurons in the *substantia nigra*, accompanied by the accumulation of Lewy bodies containing aggregated α-synuclein [10]. On the other hand, the main feature of ALS is the degeneration of motor neurons and the aggregation of the transactivation response DNA-binding protein (TDP)-43 [11], while for MS demyelination with the consequent formation of sclerotic plaques and the infiltration of immune cells in the CNS is the key pathogenetic event [12]. Precipitation and deposition of aggregated and misfolded proteins further stimulates glial cell reactivity, generating an auto-amplifying loop of detrimental events which eventually promote neurodegeneration.

For these reasons, glia reactivity and subsequent neuroinflammation represent common features shared by neurodegenerative diseases; they are triggered not only by protein aggregates, but also by various types of brain insults (e.g., ischemic stroke and traumatic brain injury (TBI)) and painful stimuli. As mentioned above, glia contribute to the pathophysiology of neurological disorders by exerting both protective and detrimental effects. Glial cells respond to external signals associated with any form of CNS pathology by undergoing complex and variable changes in their morphology, molecular expression and function, thus leading to the development of CNS disorders [13]. It has been well documented that, under pathological conditions, microglia and astrocytes become activated and release a wide range of pro-inflammatory mediators, such as IL-1β, IL-6, TNF-α, reactive oxygen species (ROS) and nitric oxide (NO). Chronic glial activation could therefore exacerbate and sustain a pathological condition through the release of excessive amounts of these cytotoxic factors, thus initiating a damaging cascade that leads to impaired neuronal function and death [7,9]. The role of glial cell activation in various neurodegenerative disorders is summarized in [9].

The rising interest in the role of glia in neurodegenerative disorders comes from the awareness that the incidence of these pathologies is increasing worldwide due to better life expectancy, but their pharmacological management is particularly challenging. In fact, effective therapies that specifically alter the pathophysiology of neurodegeneration are currently lacking, and most pharmacological treatments available to date aim primarily to control the associated symptoms rather that the pathogenesis of the disease itself. Since these diseases are characterized by multiple symptoms, the use of multiple drug treatments is sometimes necessary, with high dosages that could lead to serious side effects and make the management of the condition progressively more difficult (see [14,15,16] for current pharmacotherapy of the main neurodegenerative disorders).

Given the role of glial cells in the pathogenesis of neurodegenerative diseases, turning off the common neuroinflammatory basis by modulating these cell populations could be a potential therapeutic strategy for the treatment of brain disorders. An exponentially growing number of published papers on *in vitro* and pre-clinical models of neurodegenerative disorders has identified several possible glial pharmacological targets (see Table 1 for a summary, not comprehensive, of the most interesting) to be further exploited in humans.

As for the development of any other new therapy, and also for new glia-focused strategies to neurodegeneration, the bottleneck is represented by the translation of data to a clinical setting. In this respect, the first fundamental step is understanding if glial cells are as actively participating in pathological conditions in humans as they have been demonstrated to participate in animals.

Within this framework, the aim of this review is to summarize the contribution of glial cells to human neurodegenerative diseases and their possible pharmacological modulation based on data in *post-mortem* tissues and in iPSC-derived human brain cells and organoids. Results obtained with these innovative techniques will hopefully set the basis for the development of new pharmacological tools targeting glial cells, which could also be exploited in other brain pathologies (e.g., schizophrenia, depression, anxiety) in which a role for glial cells is also emerging.

## 2. Involvement of Glial Cells in Neurodegenerative Processes: Data from *Post-Mortem* Human Tissues

Several decades of research in animal models of brain pathologies have undoubtedly demonstrated the key roles played by glial cells in driving and sustaining brain disorders. When translating these pre-clinical data to humans, it is nevertheless important to consider that the undeniable higher complexity of the human with respect to the rodent brain is also reflected in glial cells. For example, studies in human brain tissues from surgical resections have shown that cortical astrocytes are more than 2-fold larger in diameter, extend 10-fold more GFAP-positive primary processes and spread faster calcium waves than rodent ones and are organized into specific domains which are not observed in rodents [59]. It can be, therefore, easily hypothesized that data obtained in rodent models of pathology reflect a minimal percentage of the possible connections and activities maintained by human glial cells.

*Post-mortem* tissues from patients who died from neurodegenerative disorders represent the first logical approach to clarify if glia cell reactivity represents a hallmark and a driver of neurodegeneration in humans as well as in rodents. Nevertheless, as mentioned above, the classical dualistic “Dr. Jekyll and Mr. Hyde” vision of microglia and astrocyte reactivity, with one overtly detrimental status counteracted by a fully protective one, has currently been overcome by a more dynamic and blended equilibrium among different conditions in which morphological characteristics do not always correspond to specific and distinct functional correlates. Thus, it is very difficult to drive any functional conclusion based only on the morphological changes that can be observed in fixed tissues. Whatever its functional meaning is, signs of glia reactivity have indeed been demonstrated in human brain pathologies.

In the entorhinal cortex of human AD brains, the altered expression of proteins associated with defective synaptogenesis [i.e., HSP90AA1, PTK2B, and ANXA2] has been observed to be localized with microglia and astrocytes in close proximity to Aβ plaques [60]. Single-cell RNA-seq transcriptomic analysis further confirmed that cell-specific markers of glial cells are upregulated in AD brains with respect to control tissues [61]. An increased number of microglia cells with an activated amoeboid shape was also detected in the midbrain of post-mortem PD tissues with respect to controls. Single-cell transcriptomic analysis showed a dysregulation of genes related to unfolded protein response and cytokine signaling, with a specific pro-inflammatory trajectory and overexpression of *IL1β* and other mediators. Astrocytes showed an overexpression of *CD44* and abnormal disease-related proliferation [62]. The overexpression of the glia maturation factor [GMF], a pro-inflammatory protein that has been demonstrated to orchestrate the immune cell–neuron–glia crosstalk *in vitro* and in animal models of PD [63], was also observed in the *substantia nigra* and *corpus striatum* of human PD brains, in close contact with areas of neuronal degeneration [64].

## 3. A Window to Human Glial Cells Reactivity: *In Vivo* Monitoring of Neuroinflammation

In animal models of brain pathologies, live imaging of glial cell reaction to injury and neurodegenerative conditions can be quite easily achieved thanks to the use of fluorescent tracers or to the generation of transgenic animals in which specific brain cell populations are tagged to be visualized live under two-photon microscopes. PET (Positron Emission Tomography, a functional imaging technique that uses radioactive tracers to visualize and measure changes in physiological processes) analyses have also been set up, which, at variance from fluorescence imaging, could later be translated to the clinics.

Great expectations for the direct, dynamic and *in vivo* evaluation of glial cell activation in humans came from the discovery of the 18 kDa translocator protein, named TSPO, a five-transmembrane domain protein expressed by mitochondria in different cell types but, interestingly, specifically upregulated in astrocytes and microglia upon their activation [65]. TSPO expression can be monitored by PET thanks to several radioligands, as recently reviewed in [66], including the ^11^C-PBR28 radioligand, whose accumulation showed significant glial activation in the brains of animal models of pain and of patients suffering from low back pain, migraine, fibromyalgia and other painful conditions but also neurodegenerative disorders characterized by extensive neuroinflammatory processes, such as Huntington’s disease and MS [67,68,69]. The molecular mechanisms leading to TSPO upregulation in glial cells have not only been identified, but overall TSPO monitoring is currently considered as a reliable marker for the development of neuroinflammatory processes.

Although generally considered as a marker of neuroinflammation, a major limitation of tracers targeting TSPO is that they cannot differentiate between pro- and anti-inflammatory glia phenotypes. Thus, new tracers with an additional discriminating ability are needed. In this respect, a new PET radiotracer, named [^18^F]OP-801, which is selectively taken up by phagocytic microglia and macrophages in the brain is currently on the way to its first-in-human phase I/II clinical trial for the monitoring and evaluation of neuroinflammation in patients [70]. Additionally, a number of proteins expressed by microglial cells are currently under evaluation for their reliability as markers of microglia activation and for the possibility of being visualized thanks to selective radioligands [66]. For example, the [^11^C]KTP-Me and [^11^C]PS13 ligands have been demonstrated to bind to COX1; conversely, [^11^C]MC1 is selective for the COX2 isoform [71,72]. Indeed, various ligands targeting the P2X7 purinergic receptor subtype have been developed, including [^11^C]JNJ-54173717 (JNJ-717), [^18^F]JNJ-64413739, and [^11^C]SMW139 [66]. The expression of the P2X7 receptor has been found to be upregulated in various neurodegenerative disorders [73], and could therefore represent a reliable marker of pathological glia activation.

One important aim of research would be the disentanglement of the role of reactive microglia and astrocytes in human brain pathologies thanks to the visual discrimination of the two cell populations. Despite the intrinsic difficulties in finding really specific targets to be monitored, several proteins, including the monoamine oxidase B (MAO-B) enzyme and the mitochondrial imidazoline2 binding sites (I2BS), or metabolites (acetate) are currently emerging as astrocytic-specific markers of activation and are targeted by selective PET tracers [66]. On the www.clinicaltrials.gov website (accessed on 8 March 2024), a list of several in progress clinical trials aimed at monitoring astrocytic activation in patients affected by neurodegenerative disorders, including AD and PD, can be found.

## 4. Development of Innovative Methods to Study Functional Human Glial Cells

**Figure 1:**
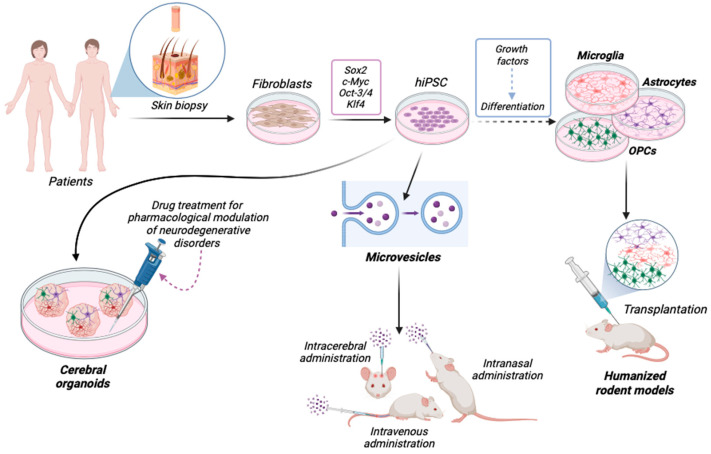
Human induced pluripotent stem cells (hiPSCs) derived from patient’s somatic cells, particularly from human fibroblasts obtained via skin biopsy, can be differentiated into a variety of cell types, including astrocytes, microglia and oligodendrocyte precursor cells (OPCs). hiPSCs have multiple potential applications in preclinical research, including generation of brain organoids and humanized mouse models by cell transplantation, and can be used as drugs themselves by directly administering iPSC-derived microvesicles to rodent models of disease. See text for details. Created with BioRender.com (accessed on 18 March 2024).

Analyses on *post-mortem* or surgery-derived tissues and even the direct *in vivo* evaluation of glia activation do not necessarily provide a satisfactory answer to the fundamental question of “the chicken or the egg”, i.e., is glia activation directly responsible for the progression of the neurodegenerative processes or is it rather a consequence of neurodegenerative triggers? This question is not trivial, since its answer is fundamental to understand if limiting glia activation with pharmacological approaches could slow down neurodegenerative disease progression or, conversely, could paradoxically bear overall negative consequences. Thus, new ways of studying the characteristics and behavior of living human glial cells have been identified thanks to induced pluripotent stem cells (iPSC) and organoids technologies, paralleled by the development of humanized mouse models of brain disorders (Figure 1).

### 4.1. Human iPSCs

iPSCs are self-renewable cells derived from somatic cells, typically fibroblasts from human skin biopsy, that can be re-programmed to generate several cell types via different methods [74]. Their advantage over preclinical animal models of diseases is that iPSCs more accurately represent the human genome, so they can be used for both human disease modelling and drug discovery. iPSCs represent a powerful tool to study human CNS cells *in vitro* since, so far, they have been successfully differentiated into neurons, astrocytes, microglia and oligodendrocytes from both healthy subjects and patients [75]. Additionally, patient-derived cells directly mirror his/her specific genetic, epigenetic and clinical characteristics. Thus, they can be extremely useful to study interindividual variabilities to drug action and to develop true personalized therapies, or in monogenic diseases their genome can be modified using gene editing technologies with “corrected” cells in turn transplanted to target tissues [75]. To date, most studies have used human iPSCs as a tool to dissect the molecular and cellular mechanisms underlying CNS (and other) diseases, generating the so-called “disease-in-a-dish”. However, very recent studies have begun to highlight a potential therapeutic role of the pharmacological modulation of glial cells aimed at generating a neuroprotective milieu which could prevent neuronal damage, also helping to clarify relevant species-related differences. For instance, human iPSC-derived microglia exposed to LPS displayed a metabolic shift, as already reported for the immortalized mouse microglial cell line BV-2 [76], and an overall increased glycolytic gene signature. At variance from mouse microglia, which in response to LPS treatment showed a metabolic reprogramming characterized by the upregulation of hexokinases, human microglia displayed upregulated phosphofructokinases, highlighting the species-specificity of the pathways involved in immunometabolism and the importance of considering these differences in translational research [77]. Acute exposure of human iPSC-derived microglia to IL-6, which at prenatal stages is associated with increased risk for psychiatric disorders, resulted in STAT3 phosphorylation and increased *IL6*, *JMJD3* and *IL10* gene expression, indicating the activation of IL-6Ra signaling. In addition, acute IL-6 stimulation increased microglia motility and induced microglial cells to secrete a number of pro-inflammatory mediators. Interestingly, RNAseq analyses identified multiple up-regulated genes in IL-6-exposed microglia that overlapped with an up-regulated gene set from human post-mortem brain tissue from patients with schizophrenia, indicating that IL-6-induced microglia activation contributes to mimic functional phenotypes of relevance for psychiatric disorders [78]. The role of microglia was also investigated in frontotemporal dementia linked to chromosome 3 (FTD3), a rare sub-form of the disease caused by a point mutation in the gene encoding for Charged Multivesicular Body Protein 2B (CHMP2B), by implementing healthy control iPSCs with either a heterozygous or homozygous *CHMP2B* mutation. iPSCs were next differentiated to microglia to evaluate their pro-inflammatory profile and metabolic state, while iPSC-derived neurons were cultured with microglia conditioned medium to investigate disease-specific interactions between the two cell populations. Authors identified two distinct microglial phenotypes resulting from the underlying mutations: a severe pro-inflammatory profile in *CHMP2B* homozygous FTD3 microglia and an “unresponsive” microglial state triggered by *CHMP2B* heterozygous FTD3. Conditioned medium from *CHMP2B* homozygous FTD3 microglia caused neurotoxic effects, which was not observed for heterozygous microglia. Surprisingly, IFN-γ treatment initiated an immune boost of the *CHMP2B* heterozygous FTD3 microglia, and conditioned microglia media exposure promoted neural outgrowth, suggesting that the heterozygous state of the mutation in FTD3 patients could be potentially exploited as an immune-boosting intervention strategy to counteract neurodegeneration [79]. Another study used single-cell RNA sequencing to measure the transcriptional response of iPSC-derived microglia after 24 and 48 h of stimulation with prostaglandin E_2_ (PGE_2_) or LPS+IFN-γ as pro-inflammatory and microglia-priming stimuli, either alone or in combination with ATPγS to see whether they enhanced microglia response to ATP. Authors observed a shared core transcriptional response of iPSC-derived microglia to ATPγS and to LPS+IFN-γ, suggesting a convergent mechanism of action, while the expression profiles of PGE_2_-treated cells were more similar to those of untreated control cells. Differentially expressed genes in iPSC-derived microglia across all treatments significantly overlapped with genes that change in the microglia of AD patients. Moreover, authors identified a common axis of transcriptomic change across microglia from genetic mouse models of AD, and showed that LPS treatment alone is able to shift the transcriptional profile of human iPSC-derived microglia towards a disease state [80]. This observation is relevant since, although LPS is not known to cause AD, the Toll-like receptor 4 that mediates LPS response is thought to have a role in the disease [81].

As for astrocytes, it is known that their reactivity is involved in the pathogenesis of AD by ingesting large amounts of Aβ, which leads to severe cellular stress. Human iPSC-derived astrocytes were exposed to sonicated Aβ42 fibrils and the direct and indirect effects of the Aβ-exposed astrocytes on human iPSC-derived neurons were analyzed by astrocyte–neuron co-cultures and by exposure of neuronal cultures to astrocyte-derived conditioned media or extracellular vesicles. Electrophysiological recordings revealed a significantly decreased frequency of excitatory post-synaptic currents in neurons co-cultured with Aβ-exposed astrocytes. Moreover, factors secreted from control, but not from Aβ-exposed astrocytes, had a beneficial effect on neuronal cultures, and reactive astrocytes with Aβ deposits led to an elevated clearance of dead cells in the co-cultures, showing that the inclusion of aggregated Aβ affects the reactive state of the astrocytes, as well as their ability to support neuronal function [82]. Another study investigated iPSC-derived S100β-positive glial cell cultures from healthy donors and from PD patients with *PARK2* mutations under resting conditions and upon stimulation by TNF-α. Non-stimulated glia from PD patients showed higher *IL1β* and *IL6* expression levels and increased IL-6 protein synthesis compared to glial cells from healthy donors. Conversely, TNFα-stimulated glial cultures from both PD patients and healthy donors displayed an increased expression of genes encoding for pro-inflammatory cytokines, although PD glia responded to TNF-α stimulation less strongly than healthy glia. Authors assumed that glial cells in *PARK2*-associated PD have a “more inflammatory” status in the resting state but respond less strongly than healthy glia to inflammatory challenges, suggesting a reduced activation capacity [83]. Human iPSC-derived neurons and astrocytes were exposed to pro-inflammatory cytokines (i.e., TNF-α and IL-17A) typically associated with progressive multiple sclerosis (PMS). Increased neurite damage was observed in neurons from both progressive MS and benign MS (BMS) patients, the latter being a form of relapsing-remitting MS with very mild or no attacks separated by long periods with no symptoms [84] and used as control. In contrast, TNF-α/IL-17A-reactive BMS astrocytes cultured with healthy control neurons exhibited less axonal damage compared with PMS astrocytes. Accordingly, the single-cell transcriptomic analysis of neurons co-cultured with BMS astrocytes revealed upregulated neuronal resilience pathways, and supernatants from BMS astrocyte/neuronal co-cultures rescued TNF-α/IL-17A-induced neurite damage [85]. Despite the marginal role of oligodendroglia in neuroinflammation and neurodegeneration, the transplantation of iPSC-derived oligodendrocyte progenitor cells (OPCs) at the injury site was recently developed as a potential therapeutic strategy for promoting remyelination [86] and, consequently, locomotor function recovery in CNS disorders such as MS and spinal cord injury. To this purpose, a research group demonstrated that cannabinoid receptors (CB1R and CB2R) were differentially expressed in iPSC-derived human neural stem cells (NSCs) and OPCs, and they could be activated by WIN55212-2 (WIN), a potent CB1R/CB2R agonist, to upregulate the endocannabinoid signaling during glial activation. WIN primed NSCs to generate more Olig2+ glial progenitors and migratory PDGFRα+ OPCs in a CB1/CB2 dependent manner compared to unprimed NSCs. Furthermore, WIN-induced OPCs robustly differentiated into functional oligodendrocytes and myelinate *in vitro* and *in vivo* in a mouse spinal cord injury model, and RNA-Seq revealed that WIN upregulated the biological process of oligodendrocytes differentiation [87].

Despite the emerging potential of human iPSC-derived glia to identify novel therapeutic targets and drug candidates for complex CNS disorders and to pave the way for new opportunities for drug discovery and potentially personalized medicine in this area, pharmacological studies published so far utilize iPSCs-derived neurons. However, promising data obtained on iPSCs differentiated to glial cell populations not only represent a solid tool for disease modeling, but will also likely drive successful drug development programs.

### 4.2. Cerebral Organoids

To date, preclinical models have proved insufficient to reproduce the complexity of neurodegenerative diseases in humans. Suffice it to say that drug candidates for AD have a failure rate around 99.6% in clinical trials [88]. This indicates a strong need for improved disease models to more accurately reproduce the disease biology in humans. Despite the fact that iPSCs have revolutionized *in vitro* studies by granting access to a virtually unlimited supply of human cells, cell cultures cannot recreate the complex microenvironment of human brain tissue. 

Cerebral organoids derived from iPSCs were first described a decade ago and represent a promising novel tool for compound screening applications, although the heterogeneity and random occurrence of different brain regions in cerebral organoids limit their utility as disease models (Figure 1) [89]. The optimization of differentiation conditions and automation have recently led to the generation of more homogenous, brain region-specific organoids capable of resuming key molecular hallmarks of CNS diseases [75]. The access to patient-derived tissue provided by cerebral organoids opens up to opportunities for drug discovery. Indeed, they can be used to: (a) validate results from single-cell RNAseq studies on post-mortem tissues that identify specific molecular pathways affected by disease; (b) probe the effects of disease risk gene variants identified in population-wide studies by using gene editing techniques; (c) screen for different kinds of environmental perturbations that can promote disease and (d) integrate insights from these approaches with improved methods for high-throughput screening to yield promising drug candidates to be further validated using preclinical models and in clinical trials [90].

To date, most brain organoid models are predominantly composed of different neuronal types, with a smaller percentage of glial cells compared to the real composition of brain tissue [91]. Therefore, improving their cellular composition to be more representative of the physiological status of the brain is crucial to ameliorate 3D *in vitro* human systems. In this respect, an interesting work showed that integrating human iPSC-derived microglia into iPSC-derived midbrain organoids exerts positive effects on cell death and oxidative stress-related gene expression, affects synaptic remodeling and increases neuronal excitability, overall leading to increased neuronal maturation and functionality [92]. Another study implemented a chemically defined glial-enriched medium (GEM) to expand the population of astrocytes and oligodendrocytes without compromising neuronal differentiation in brain organoids. GEM enhanced neurite outgrowth and cell migration, and modulated neuronal maturation, showing its potential to significantly improve the functionality of brain organoids for the study of neurological diseases and drug discovery [93]. Very recently, a glia-enriched cortical organoid model displayed accelerated astrogliogenesis. By triggering a gliogenic switch in 28–33% of the cells in the organoids at 3 weeks of differentiation, the authors achieved an efficient derivation of astroglia comprising 25–31% of the cell population by 8–10 weeks of differentiation. Moreover, after intracerebral transplantation, organoid-derived cells displayed robust integration into the host brain and developed anatomically defined morphological subclasses of human astrocytes, and differentially expressed genes associated with acute reactivity exhibited significant heterogeneity across astrocyte subpopulations in an *in vivo* model of acute neuroinflammation. Moreover, in this model, the authors demonstrated that metabolic and mitochondrial stress in reactive astrocytes is mediated by CD38 signaling and that treatment with a potent CD38 inhibitor effectively alleviated a wide range of stresses induced by inflammation in astrocytes [94].

Another research group generated microglia-enriched brain organoids by coculturing brain organoids with primitive-like macrophages generated from the same human iPSCs. In organoid co-cultures, macrophages differentiated into cells with microglia-like phenotypes and functions and modulated neuronal progenitor cell differentiation, limiting their proliferation and promoting axonogenesis. The authors observed that these microglial cells contained high levels of PLIN2+ lipid droplets that exported cholesterol and its esters, which were taken up by neural progenitor cells in the organoids. Interestingly, PLIN2+ lipid droplet-loaded microglia were also detected in mouse and human embryonic brains, showing a key pathway of lipid-mediated crosstalk between microglia and neural progenitor cells that improves neurogenesis [95]. Overall, although there are no studies involving the pharmacological modulation of glial cells in brain organoids, published literature data demonstrate the crucial role of this cell population in the development of 3D models of diseases for their future application in drug discovery.

### 4.3. Humanized Mouse Models

The successful use of human iPSCs to model diseases in 2D and 3D cell culture paved the way for their use to obtain rodent models of neurodegenerative diseases that more closely resembled the human condition, i.e., “humanized” animal models (Figure 1).

Human iPSCs differentiated into microglia precursors were transplanted to the lateral ventricles of immunodeficient neonatal mice also carrying human transgenes for *CSF1*, *IL3*, *KITL* and *CSF2*. Precursors efficiently turned into mature microglia, bearing characteristic microglial morphology and gene expression signatures that closely resembled primary human microglia. Moreover, the single-cell RNA-sequencing analysis of transplanted microglia showed similar cellular heterogeneity as primary human cells. When transplanted mice were stimulated with LPS, microglia cells switched to an activated state, demonstrating that the transplantation of human microglial progenitors to the mouse brain represents a potential model for studying the activation of human microglia in the brain [96].

Another study showed similar results: the authors also transplanted microglia precursors derived from human iPSCs into immunodeficient mice expressing human *CSF1* at postnatal day 0. At 6 months post-transplantation, mature microglia cells with a genetic profile closely resembling human microglia were found throughout the mouse brain, and showed that they pruned synapses, contacted blood vessels, and phagocytosed damaged oligodendrocytes. Interestingly, the gene expression profile of these microglia revealed that they express human neurodegenerative disease-relevant genes differentially to mouse microglia. Of note, the engrafted iPSC-derived microglia exhibited a dynamic response to cuprizone-induced demyelination and upregulated the expression of genes also observed in MS patients [97].

In the field of CNS diseases, to date only a few published papers have used iPSC transplants to obtain humanized AD mouse models, with the first study published in 2017 [98]. More recently, human iPSC-derived microglia have been transplanted to an AD mouse model. In 2019, Hasselmann and colleagues transplanted microglia progenitors to postnatal day 1 humanized immunodeficient mice. Transplanted cells differentiated into mature microglia, resembled human microglia in their transcriptomic profile, acquired different morphologies depending on their brain location and became activated after exposure to LPS. Therefore, the authors used a genetic AD mouse crossed with humanized immunodeficient mice to study how the transplanted human microglia responded to Aβ plaques. Microglia response to Aβ fragments was characterized by a downregulation of the purinergic P2Y_12_ receptor and upregulation of markers associated with microglial response to disease, as well as by an amoeboid shape and the ability to phagocytose the fibrillar form of the protein. Transcriptomic analysis also revealed a differential gene expression profile that was specific to the human microglia response to Aβ, showing that the transplantation of microglia derived from human iPSCs to a mouse model of neurodegenerative disease has the potential to give insight into the human condition [99].

From a pharmacological point of view, although to date no new therapeutic entities have been identified, humanized mouse models represent important preclinical tools with great potential application in scientific translation. Indeed, as suggested by published literature, they could serve as tools to elucidate drug response and safety, as well as for druggable target identification.

## 5. Glial Cells as Drugs Themselves: Administration of Glia-Derived Microvesicles

The most logical approach to any pathology characterized by cell death and tissue degeneration would theoretically be the transplantation of new cells that later integrate into the damaged environment, replenish the lost cell populations and reconstitute the injured physiological connections and, eventually, tissue and organ functionality. In real life, however, this strategy has proved extremely difficult to be exploited with successful results even in animal models of brain diseases. This is mostly due to the difficulty in finding the right cell population to be transplanted without leading to immune cell reaction and cell rejection, to the low survival and limited integration of transplanted cells at the injured site also due to an unfavorable pro-inflammatory environment, and to the overall high risk of the development of secondary tumors [100]. Difficulties dramatically increase when protocols for humans are set up, not only from a biological point of view but also due to the stringent ethical concerns that arise, for example, from the hypothesized use of human embryonic cells. Nevertheless, phase I and II clinical trials are currently ongoing in ALS and MS [101].

An alternative emerging strategy could be represented by the exploitation of one particular route of communication utilized by many cell populations not only in the CNS, i.e., the release of extracellular vesicles (EVs). EVs are classified based on their dimensions, are delimited by a double layer membrane, contain a huge variety of cell components, including nucleic acids, proteins, small interfering RNAs, neurotransmitters, lipids and others [102], and are produced under physiological conditions by virtually all cells in the body to promote cell-to-cell communication. Since they are often released in the bloodstream and in other circulating fluids, including the cerebrospinal fluid, their effects can be observed at a distance from the cell of origin. 

As far as glial cells are concerned, astrocytes physiologically secrete EVs that represent crucial players in maintaining normal neuronal functions, including promoting neurite outgrowth and neuronal survival. Also, microglia-derived EVs participate in various physiological functions, including the metabolic support of neurons and synaptic activity and transmission, as well as neuronal survival. Interestingly, glia-to-glia, including microglia-to-astrocyte, cell communication can be modulated by the reciprocal release of EVs as well [103]. Interestingly, pathological conditions, including neuroinflammation, significantly alter the amount and the cargo of both astrocyte- and microglia-derived EVs, so that their final outcome could be protective or inflammatory depending upon the activation status of generating cells [104]. For example, EVs secreted by astrocytes exposed to pro-inflammatory TNF-α or IL-1β or by LPS-treated microglia have proved detrimental to neuronal cells, with the opposite effect when EVs were spread by glial cells exposed to neuroprotective and anti-inflammatory cues, such as IL-4 [103].

Interestingly, original evidence sustained an overall beneficial role of glia-derived EVs in neurodegenerative disorders since they were demonstrated to contribute to the clearance of damaged cells and pathological proteins. Conversely, more recent data suggest that under several pathological conditions EVs can contribute to spread neurodegenerative signals, as demonstrated in MS and other demyelinating diseases [105]. Thus, the role of EVs in brain disorders is extremely complex, as reviewed in [103], and, consequently, the strategies toward their possible therapeutic use face significant challenges.

Nevertheless, several studies are currently reporting potential beneficial effects of the administration of EVs derived from glial cells, iPSCs or other types of stem cells, including human mesenchymal stem cells, in *in vitro* and animal models of neurodegeneration. Just to mention some examples, EVs derived from stem cells isolated from the dental pulp of human exfoliated deciduous teeth have demonstrated significant anti-apopotic effects both *in vitro*, on dopaminergic neurons exposed to neurotoxic insults mimicking PD, and via intranasal *in vivo* delivery, to a rat model of the pathology [103]. EVs derived from astrocytes exposed to acidic fibroblast growth factor have proved beneficial in a mouse model of AD [106], whereas EVs isolated from microglia cells exposed to an anti-inflammatory stimulus (e.g., IL-4) improved post-stroke recovery in mice subjected to middle cerebral brain artery occlusion (MCAo) [107], and EVs from stem cells and astrocytes positively modulate functional outcomes after TBI [108]. 

Moving to humans, EVs are currently under consideration as extremely interesting potential biomarkers for brain diseases, brain disease progression and/or response to a given therapy since they can be isolated from the blood or from other easily accessible body fluids and carefully mirror CNS status [109]. As far as their therapeutic potential is concerned, once again the translation to the clinic of data obtained in animals poses significant challenges that are first of all related to the correct comprehension of the beneficial/detrimental role of endogenous EVs in a specific pathology and of the possibility to modulate pathological pathways through the administration of exogenous EVs whose composition should be carefully analyzed. Additional technical issues concerning the isolation of clinical-grade pure EVs must also be taken into consideration, as well as the most suitable route of administration to patients. Alternative strategies, including the laboratory production of artificial nanovesicles, are currently under development, so that their composition, structure and characteristics can be fully controlled and modulated according to the needs of a specific patient. Not less importantly, thanks to their ability to permeate the blood-brain barrier, EVs can also be utilized to directly deliver drugs to the CNS, as recently reviewed in [102].

## 6. Drugs Targeting Glial Cells

Table 2 summarizes currently available data on drugs that, either exclusively or (in most cases) not, target glial cells and, as a consequence, are potentially effective in brain disorders. The vast majority of results have been obtained in pre-clinical animal models, but data on humans are progressively emerging with some clinical trials already ongoing.

Astrocyte-specific therapies or drugs have not been developed so far; however, many drugs with various primary molecular and cellular targets also exert their pharmacological effects on this cell population. Astrocyte-associated molecules that represent potential therapeutic targets in different disease contexts are summarized in [13] (see also Table 1). In particular, it is clearly emerging that besides trying to block the detrimental effects of astrocyte activation, there is a need to design new drugs that can preserve and enhance astrocyte-mediated defenses and improve astrocyte homeostasis, thus enabling the development of the pathophysiology-based treatment of CNS diseases [13].

On the other hand, pharmacological treatments targeting the complex microglial heterogeneity are available, not merely attenuating their excessive inflammatory and phagocytic activity, but also acting on their proliferative, metabolic and surveillance functions. Although they lack the full necessary selectivity, available pharmacological strategies aimed at targeting microglial properties and functions associated with a specific disease state are currently in the preclinical or even clinical stages of study (Phase I-III). The main pharmacological approaches targeting microglia and related preclinical studies in various CNS pathologies are summarized here [165]. As detailed in Table 1 and Table 2, promising drugs and pharmacological targets include minocycline, antibodies against cytokine receptors, complement modulators, purinergic receptors, fractalkine, CSF1R and TREM2. Their modulation in different pathological settings improves brain inflammatory balance [166]. There are also alternative therapeutic approaches with a wider impact on microglial immune response (e.g., cannabinoids; Table 2). Thus, data obtained so far underline the need to find pharmacological agents that more selectively and specifically target microglia to further drive neurological drug development. 

## 7. Concluding Remarks

Overall, based on the above-mentioned considerations and on several other papers which could not be included due to space limitations, targeting glial cells to treat neurodegenerative disorders appears an appealing and innovative strategy when we consider animal rodent models in which the disease is induced experimentally, which does not necessarily guarantee a satisfactory translation to humans. The reality of this assumption is demonstrated by the failure of several clinical trials with drugs [i.e., propentofylline and the CCR2 antagonist AZD2423] to be utilized in chronic pain that should, at least theoretically, target glial cells only based on successful pre-clinical evaluations [167]. Negative clinical outcomes have unveiled that the situation is different and much more complicated when dealing with long-lasting pain conditions in patients compared to animal models. Additionally, it is quite difficult to have real “glia only” acting drugs due to the ubiquitous expression of several putative targets or to the general mechanisms of action of the drugs. Another issue to be considered is that, as mentioned, activated glial cells are not necessarily detrimental, but can also provide support to neurons and contribute to cleaning the extracellular environment of cellular debris or infectious agents. Therefore, in the end, an indiscriminate elimination or reduction of glial cells could lead to an overall worsening of patients’ conditions.

## Figures and Tables

**Table 1:** Signaling pathways activated in glial cells with a potential role in several brain disorders, including pain transmission.

Signaling Pathway	Functions	Glial Cells	Pathology	Ref
Tropomyosine receptor kinase B (TrKB)	Regulates nitric oxide release and supports neuroinflammation.	Astrocytes Microglia	MS Pain	[17,18]
Nuclear factor kappa-light-chain-enhancer of activated B cells (NFkB)	Its activation and subsequent transcription of pro-inflammatory factors triggers inflammation and neurodegeneration.	Astrocytes Microglia	AD PD MS Pain TBI ALS Ischemic stroke	[19,20,21]
JAK/STAT pathway	Regulates homeostasis in inflammatory circumstances, cellular functions that mediate innate and adaptive immunity and cytokine production.	Astrocytes Microglia	AD PD MS Pain SCI Ischemic stroke	[21,22,23,24,25]
Purinergic receptors	These are activated by extracellular nucleotides and nucleosides whose extracellular concentrations rise following tissue damage or oxygen deprivation.	Astrocytes Microglia SGCs	AD PD MS Pain ALS Ischemic stroke	[26,27]
Neurotransmitters (glutamate, GABA)	These play a critical role in maintaining the excitation–inhibition balance. Alterations in this equilibrium contribute to neurodegeneration. They also modulate the afferent transmission of nociceptive information.	Astrocytes Microglia SGCs	AD PD MS Pain ALS	[28,29,30]
CX3CL1/CX3CR1	Its deficiency is correlated with a worsening of neurodegeneration.	Microglia SGCs	AD PD MS Pain TBI ALS Ischemic stroke	[31]
Complement system	It is involved in the control of microglial functions, such as motility, phagocytosis and cytokine release. It protects the brain from pathogens and potentially harmful stimuli, such as aberrant and misfolded proteins.	Astrocytes Microglia	AD PD MS ALS Ischemic stroke	[32,33]
Triggering receptor expressed on myeloid cells 2 (TREM2)	Expressed by an activated phenotype of microglia with protective functions for the maintenance of CNS tissue homeostasis, regulation of inflammation and phagocytosis.	Microglia	AD PD MS Pain TBI ALS Ischemic stroke	[34]
PI3K/Akt pathway	It is involved in apoptosis and regulation of inflammatory responses.	Microglia	AD PD ALS Pain	[21,35]
AMP-activated protein kinase (AMPK)	It maintains steady cellular energy levels by stimulating glucose and fatty acid uptake and oxidation in the event of energy depletion.	Microglia	AD PD MS Pain	[36]
Nitric oxide (NO)	Signaling molecule synthetized by enzymes activated only in pathological conditions.	Astrocytes Microglia SGCs	AD PD MS Pain ALS	[37,38]
Mitogen-activated protein kinase (MAPK)	Serine/threonine protein kinase with significant roles in cell proliferation, differentiation and apoptosis.	Microglia SGCs	AD PD MS Pain ALS	[21,39,40,41,42]
Toll-like receptors (TLRs)	Responsible for persistent neuroinflammation	Astrocytes Microglia	AD PD ALS Ischemic stroke	[43]
NFkB activator 1 (Act1)	Triggers the production of pro-inflammatory cytokines, chemokines and metalloproteinases.	Astrocytes	MS	[44]
Sphingosine 1-phosphate (S1P1)	Regulates cellular growth, survival and differentiation by binding to specific G-protein-coupled receptors.	Astrocytes Microglia	AD PD MS Pain ALS Ischemic stroke	[45,46]
β-1,4-galactosyltransferase 6 (B4GALT6)	It synthesizes lactosylceramide (LacCer), a lipid mediator that triggers inflammation and astrogliosis.	Astrocytes	MS	[47]
Chemokine (C-C motif) ligand 2 (CCL2)	Regulates immune cell recruitment to the site of inflammation.	Astrocytes Microglia	MS Pain SCI TBI ALS Ischemic stroke	[21,48,49,50,51,52]
C-X-C motif chemokine ligand 10 (CXCL10)	Regulates the recruitment of infiltrating immune cells into CNS lesions during neuroinflammation.	Astrocytes Microglia SGCs	AD MS Pain TBI	[53,54,55]
Vascular endothelial growth factor (VEGF)	Supports vascular permeability and CNS damage in acute inflammatory lesions.	Astrocytes	AD PD MS Pain ALS SCI Ischemic stroke	[56,57,58]

**Table 2:** Drugs targeting glial cells, their molecular targets, mechanism(s) of action and effects observed in pre-clinical and (when available) clinical *in vivo* settings.

Pharmacological Agents	Target Glial Cells	Mechanism of Action	Pathology and Species	Ref.
Minocycline	Microglia Astrocytes Potential influence on peripheral myeloid cells, oligodendrocytes, neurons, and endothelial cells.	A tetracycline-derived antibiotic with inhibitory effects on microglial pro-inflammatory cytokine release and phagocytosis.	Rodent AD → reduces microglial recruitment and recovers cognitive performance Human AD → no effects on cognitive or functional impairments	[110,111,112,113,114,115,116,117,118,119]
			Rodent MS → effects on disease course Human MS → no effects on relapses Rodent pain → strong analgesic effect in animal models of chronic pain Human pain → mixed results Rodent ALS → slows disease progression Human ALS → worsens disease progression Rodent TBI → no effects Human TBI → mixed results Rodent ischemic stroke → promotes functional recovery by modulating microglia polarization	
Complement pathway inhibitors	Microglia	Antagonists of elements of the complement cascade, they modulate microglial state and interactions with synapses.	Rodent AD → C5aR1 antagonists reduce cognitive decline and attenuate microglial activation Rodent ALS → C5aR1 antagonists slow disease progression Human ALS → humanized C1q antibody in Phase II clinical trial Rodent TBI → C5aR1 inhibitors reduce pathology severity Rodent SCI → early administration of C5aR1 antagonists accelerate recovery Rodent ischemic stroke → phase-specific C3-blocking antibodies reduce acute injury extent	[120,121,122,123,124]
Purinergic receptors modulators	Microglia Astrocytes Satellite glial cells Effects on oligodendrocytes and neurons and on various cell types (e.g., antiaggregating effect on platelets by marketed thienopyridine and other P2Y_12_ antagonists).	Agonists and antagonists of several purinergic receptors that are involved in CNS and PNS disorders.	Rodent AD → reduced neuroinflammation and neurotoxicity Rodent PD → antagonist of A_2A_, P2X1, P2X7 and P2Y_1_ receptor subtypes decrease microglia activation and slow down disease progression Rodent MS → activation or blockade of P2X4, P2X7 and P2Y_12_ modify disease course Rodent pain → antagonists at P2X and P2Y and agonists at A_3_ receptor subtypes have positive effects on different pain types Rodent ALS → antagonism of P2X7 may be beneficial at late pre-symptomatic stages Rodent TBI → Inhibition of P2X7 improves pathology outcomes, reducing microglial activation Rodent ischemic stroke → P2Y_12_ antagonists exert neuroprotective and anti-inflammatory effects. Inhibition of microglial phagocytosis by selective P2Y_6_ inhibitor aggravates neurological functions.	[125,126,127,128,129,130,131,132]
Fractalkine signaling inhibitors	Microglia Influence on peripheral myeloid cells and oligodendrocyte precursor cells.	Antagonists of CX3CR1, they act on various microglial functions (i.e., modulation of neurotransmission, neurotrophic support and regulation of inflammatory response.)	Rodent SCI → CX3CR1 inhibitors facilitate early recovery Rodent ischemic stroke → CX3CR1 antibody alleviates cognitive impairment, neuronal loss and myelin deficits	[133,134]
TREM2 agonists	Microglia Influence on peripheral myeloid cells.	They act on a receptor of the immunoglobulin superfamily that regulates microglial survival, proliferation, phagocytosis and metabolic state.	Rodent AD → enhance microglia functions and reduce amyloid pathology Human AD → Phase II and III clinical trials Rodent MS → accelerate myelin debris removal by microglia Rodent TBI → alleviate neural damage	[135,136,137]
Cannabinoids	Microglia Astrocytes	Agonists at cannabinoid receptors CB1R and CB2R, whose activation reduces pro-inflammatory cytokine production and promotes cell migration.	Rodent PD → reduction of glial activation and protection of dopaminergic neurons Rodent AD → reduction of oxidative stress and neuroinflammation Rodent MS → reduction of the clinical severity of the pathology and decrease of microglia activation Rodent pain → non-selective CB1/2 agonist reduces neuropathic pain and microglial activation Rodent ALS → CB2R agonist improves motor function and reduces microglial activation Rodent TBI → selective CB2 agonist protects white matter and drives microglial polarization toward a protective phenotype Rodent ischemic stroke → controversial results	[138,139,140,141]
Colony stimulating factor 1 receptor (CSF1R) inhibitors	Microglia Potential effects on astrocytes and peripheral immune cells.	They act on a receptor tyrosine kinase required for the development, maintenance and proliferation of microglia.	Rodent AD → inhibition of microglial proliferation and prevention of disease progression Rodent PD → reduction of microglial proliferation and protection against neuroinflammation and dopaminergic neurodegeneration Rodent MS → attenuation of microglial activation, blockade of axonal damage and neurological impairments Rodent pain → elimination of microglia and reduction of inflammation Rodent ALS → slow down disease progression by reducing microgliosis Human ALS → in Phase II and III clinical trials Rodent SCI → reduction of microglial proliferation and improvement of motor recovery Rodent TBI → microglia depletion and decreased inflammation Rodent ischemic stroke → neuroprotective effect by inhibiting microglia polarization	[142,143,144,145,146,147,148,149]
S1PR inhibitors (fingolimod, siponimod)	Astrocytes General effects on immune cells (i.e., in MS they maintain lymphocytes within lymph nodes thus limiting penetration in the CNS). Already on the market as first oral therapy for MS.	They inhibit the inflammatory responses in the brain by acting on S1PRs, principally S1PR1, and are involved in multiple processes including cell survival, proliferation, differentiation and migration.	Rodent AD → beneficial effects on AD progression by regulating neuroinflammation Animal PD → neuroprotective effect Rodent MS → reduced astrogliosis, demyelination and axonal loss, and improved pathology Human MS → Fingolimod: approved immunosuppressive therapy for RRMS Siponimod: efficacy in Phase III clinical trial Rodent pain → antinociceptive effects in multiple models of peripheral inflammation/injury Rodent ALS → protective and beneficial effects accompanied by a modulation of microglial activation and innate immunity Human ALS → Phase IIa clinical trial Rodent SCI → improved functional recovery by reducing reactive astrogliosis Rodent TBI → attenuation of glia activation Rodent ischemic stroke → reduced lesion size and improved neurological function, decreasing glia activation Human ischemic stroke → effects on a pilot clinical trial	[150,151,152,153,154,155,156,157,158,159]
B4GALT5/6 inhibitors	Astrocytes	They inhibit the synthesis of lactosylceramide (LacCer), which in astrocytes acts in an autocrine way, triggering a transcriptional program that promotes neurodegeneration and controls the recruitment and activation of microglia.	Rodent MS → suppress CNS innate immunity and neurodegeneration and interfere with astrocyte activation	[47]
Montelukast	Microglia	Leukotriene receptor antagonist, already on the market for asthmatic patients.	Rodent AD → effect on β-amyloid-induced neurotoxicity with a reduction of pro-inflammatory factors Human AD → two ongoing phase II placebo-controlled clinical trials Rodent PD → attenuation of microglial activation and protective effect on motor function deterioration Human PD → ongoing Phase II unblinded clinical study Rodent pain → attenuates neuropathic pain Rodent TBI → attenuates chronic neurological damage caused by neuroinflammation Rodent ischemic stroke → influences microglia phenotype and improves functional recovery	[160,161,162,163,164]
